# Radiographic anatomical relationship between spinous process and pedicle in thoracolumbar and lumbar spine

**DOI:** 10.1097/MD.0000000000006732

**Published:** 2017-05-26

**Authors:** Xingang Cui, Guodong Wang

**Affiliations:** Department of Spine, Shandong Provincial Hospital Affiliated to Shandong University, Jinan, Shandong Province, China.

**Keywords:** entrance point, lumbar spine, pedicle, spinous process, thoracolumbar

## Abstract

Pedicle screws are widely used in spinal surgeries, but it remains technically demanding to place. There are numerous studies on the anatomy of pedicle; however, there is very little insight on the relationship between the pedicle and the spinous process, which is an important part of the spinal posterior column.

The aim of the study was to investigate the radiographic anatomical relationship between spinous processes and pedicles in the thoracolumbar and lumbar spine, in order to reveal a novel entrance point for pedicle screw insertion.

Sixty candidates were enrolled in this study; cases were excluded with degenerative disorders and other disorders as osteoporosis, deformity, and tumor. Radiographs and computer tomography scans between T10 and L5 were obtained on each case. The distance was measured that between the superior margin of spinous process root and the superior border, the inferior border and the axis of pedicle. In laboratory, 5 fresh cadavers were used to imitate the pedicle screw insertion.

The basic reference point was supposed as the intersection between the horizontal line of superior margin of spinous process root and the central vertical line of the superior facet. For T10 to T12, the pedicle axis was 5 mm beyond the reference point. For L1 to L4, the pedicle axis was at the reference point. At L5, the pedicle axis was 5 mm beneath the reference point. In laboratory, 80 screws were all inserted into pedicles successfully according to the newly referred entrance point.

The study reveals the radiographic anatomical relationship between the pedicle and the spinous process. The pedicle axis is around the horizontal line of the superior margin of spinous process root. It provides a new anatomic mark of pedicle screw entrance point.

## 1 Introduction

The pedicle screw was first used in posterior spinal instrumentation by Boucher in 1959^[1]^ and has been a routine technique since popularization by Roy-Camille in 1963.^[2]^ Nowadays, such a technique is widely used in the lumbar spine as well as in the thoracic spine. However, the pedicle screw remains technically demanding to place.^[3]^ The key point of the procedure is to localize the entrance point. There are several methods to choose the entrance point accurately.

There are 3 main methods to localize the entrance point in the lumbar spine. (1) Roy-Cammille et al^[2,4]^ described it 1 mm beneath the facet joint in line with the lateral margin of facet joint. (2) Magerl^[5]^ chose the intersection point between the lateral border of superior facet joint and the midline of transverse process. (3) Du et al^[6]^ located the entrance point at the top joint where the isthmus crest conversed the accessory process crest. The 3 methods used different bony marks and structures nearby pedicle to localize the entrance point.^[7]^ The accuracy has been proven by numerous reports.^[8]^

However, there is no deep insight on the relationship between the spinous process (SP) and the pedicle. The study investigates the anatomy of the spinous process and the vertebral pedicle and analyzes the relationship between them. The study is designed in order to reveal a new method to localize the entrance point of pedicle screw in thoracolumbar and lumbar (TL/L) spine.

## 2 Materials and methods

A total of 60 healthy candidates were enrolled in study. Permission to conduct this retrospective study was obtained from the Hospital Ethics Committee.

The inclusion criteria were: (1) aged between 18 and 55 years old; (2) without spinal deformity, fracture, tumor, and the others spinal disorders; (3) without osteoporosis.

The exclusion criteria were: (1) elder than 55 years old or younger than 18 years old; (2) with osteoporosis; (3) with spinal deformity, fracture, tumor, and the other spinal disorders; (4) with gigantism or midgetism.

All the candidates underwent radiographs and computer tomography (CT) scans. The scan range was between T10 and L5. CT scan using a 64 slice multi-detector CT scanner (Siemens, Erlangen, Germany). All the CT scans are taken under the breath-holding condition. The section thickness of CT scan was 2.5 mm. Three division reconstruction is rendered, including sagittal and coronal imaging.

**Figure 1:**
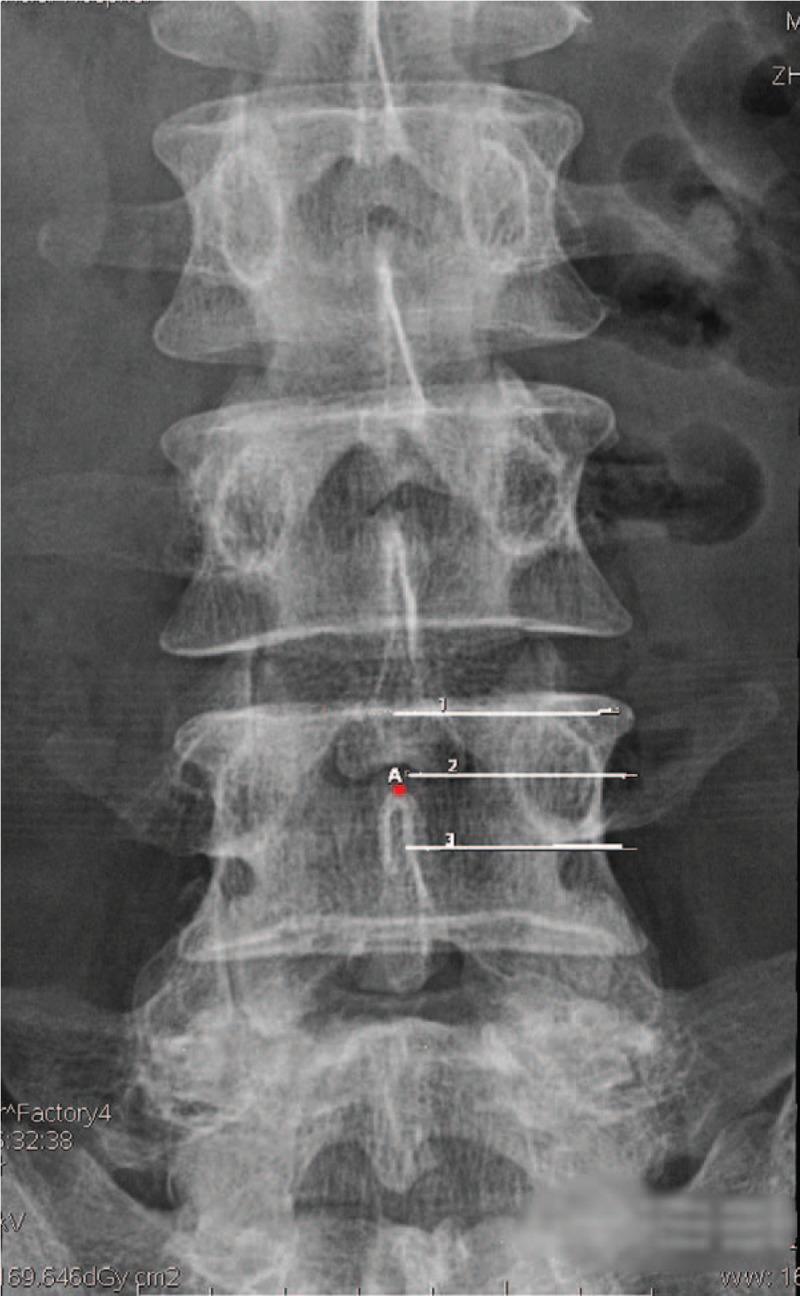
The diagram of x-ray plain films measurements. (1) The line of pedicle upper edge. (2) The line of pedicle axis. (3) The line of pedicle lower edge. Point A = the superior margin of spinous process root.

**Figure 2:**
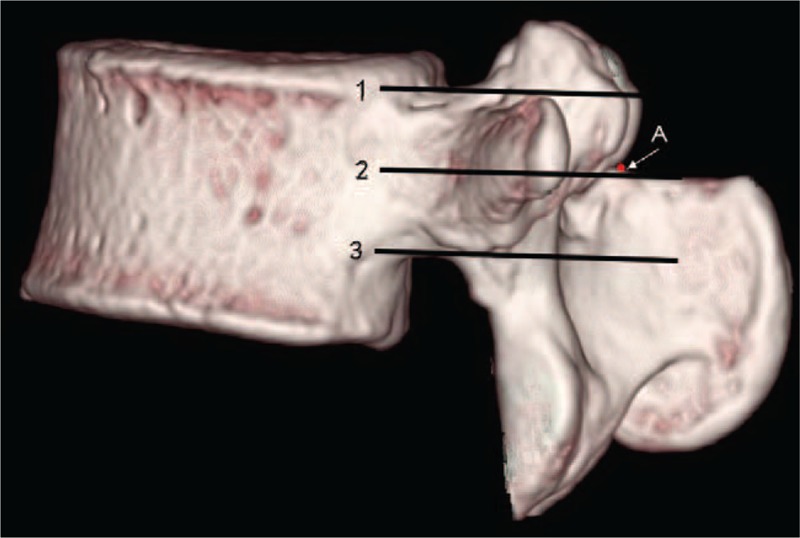
The diagram of CT scans measurements. (1) The line of pedicle upper edge. (2) The line of pedicle axis. (3) The line of pedicle lower edge. Point A = the superior margin of spinous process root. CT = computer tomography.

Radiographic measurement parameters included the vertical distances between the superior margin of SP root and the upper edge of pedicle, the lower edge of pedicle, and the axis of pedicle (shown in Figs. 1 and 2).

The collected data were analyzed. One-way analysis of variance and the LSD-t test were used for statistic comparison. *P* value less than .05 was considered statistically significant.

### 2.1 Laboratory simulation operation

**Figure 3:**
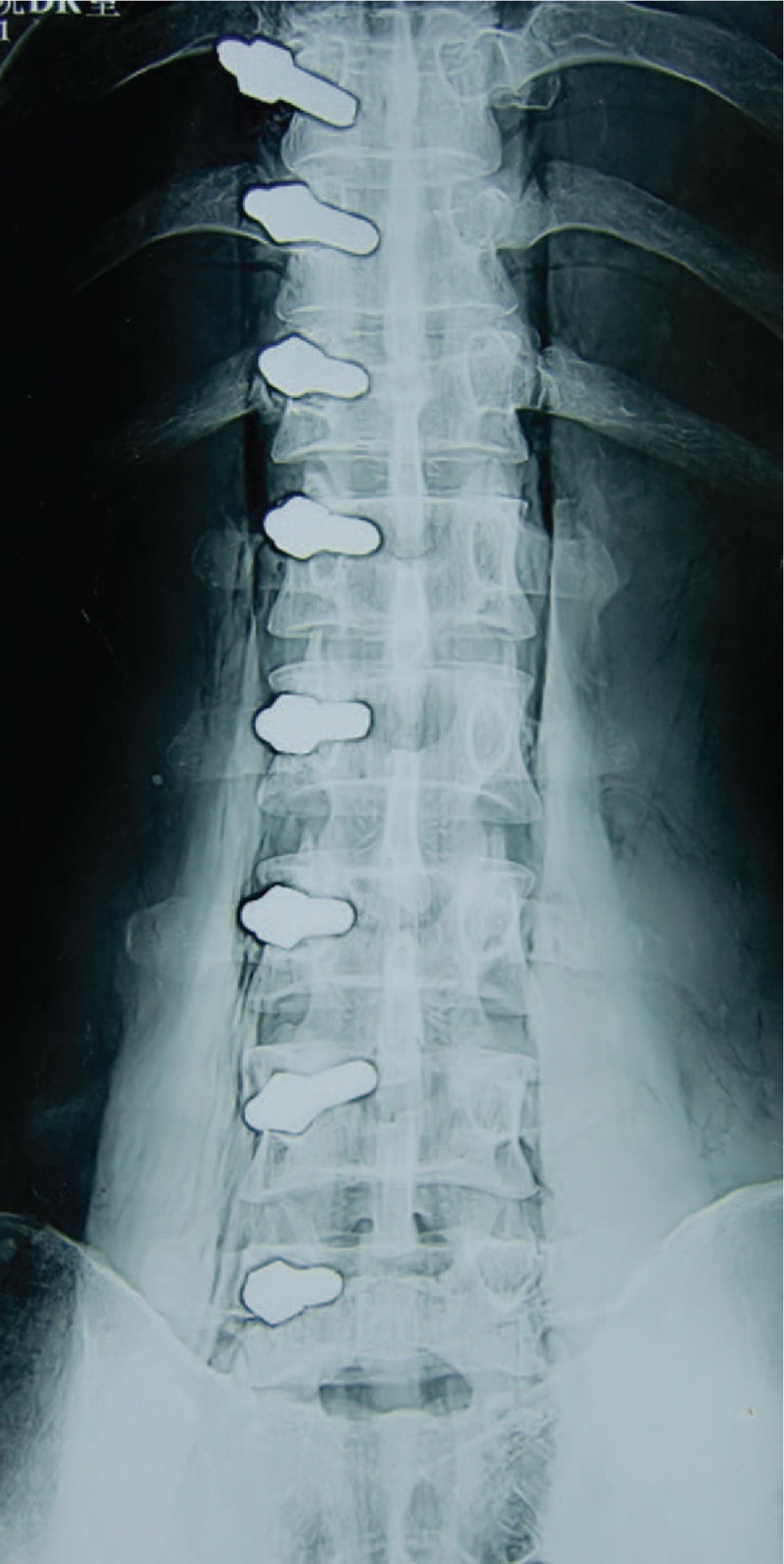
The anteroposterior radiograph after the screw placement in cadavers.

**Figure 4:**
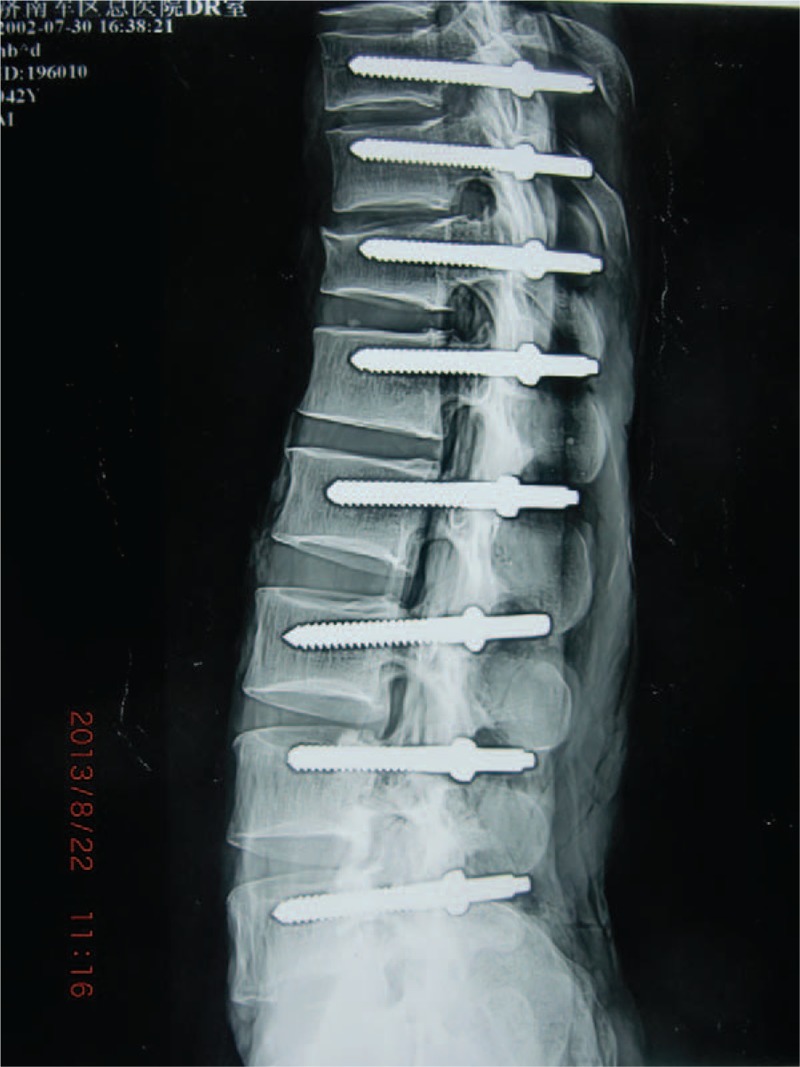
The lateral radiograph after the screw placement in cadavers.

According to the measurement results, the suitable entry point of pedicle screw was chosen based on the spinous process. The simulation screw replacement was conducted in 5 adult fresh cadavers. The placement of screw condition was observed by naked eye and under x-ray fluoroscopy (shown in Figs. 3 and 4).

## 3 Results

There were 30 males and 30 females. The average age was 37±12 years old (range 20–51 years old). The height was 167 ± 25 cm (range 155–181 cm). The weight was 61 ± 16 kg (range 45–82 kg).

It was measured that the distance between the superior margin of spinous process root and the upper edge, lower edge and axis of pedicle on radiograph and CT scan. Then, the statistic comparison was conducted between the data collected on radiograph and on the CT scan. There was no statistically significant difference between male and female. Therefore, the mean value was summarized after respective consolidation and comparisons between the 2 groups.

The distances between the upper edge of pedicle and the superior margin of SP root decreased gradually from T10 to L5. It was the longest at T10 with 16.68 ± 2.32 mm and the shortest at L5 with 0.59 ± 1.93 mm. There is no statistical significance between the CT scans and radiographs. (shown in Table 1)

**Table 1:**
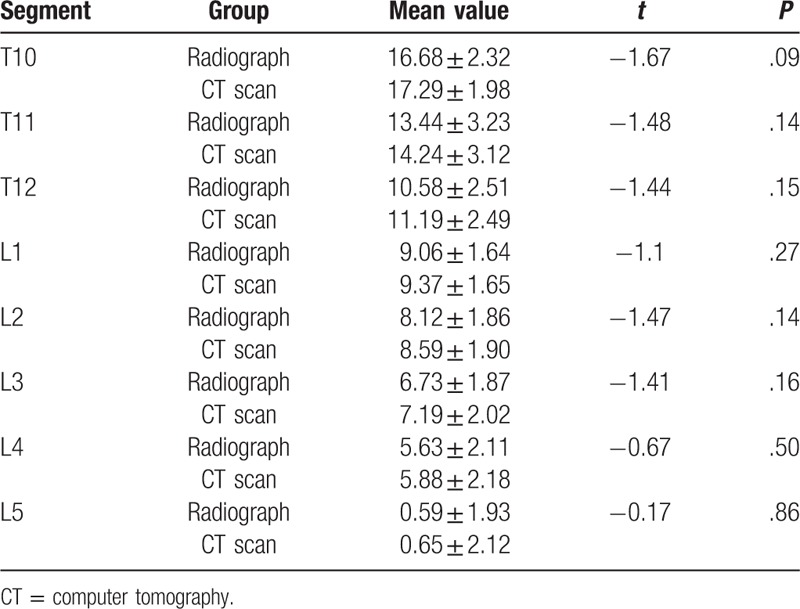
The distance between the superior margin of spinous process root and the upper edge of pedicle and comparison between radiographs and CT scans (mm *x* ± s).

The distance between the lower edge of pedicle and the superior margin of SP root increased gradually from T10 to L5 (Shown in Table 2). The superior margin of SP root at T10 and T11 were slightly lower than the lower edge of pedicle. For T12 to L5, the superior margin of SP root was all above the lower edge of pedicle. The distance ranged from 2.58 ± 2.08 mm to 9.85 ± 2.15 mm. There was no statistical significance between CT scans and radiographs.

**Table 2:**
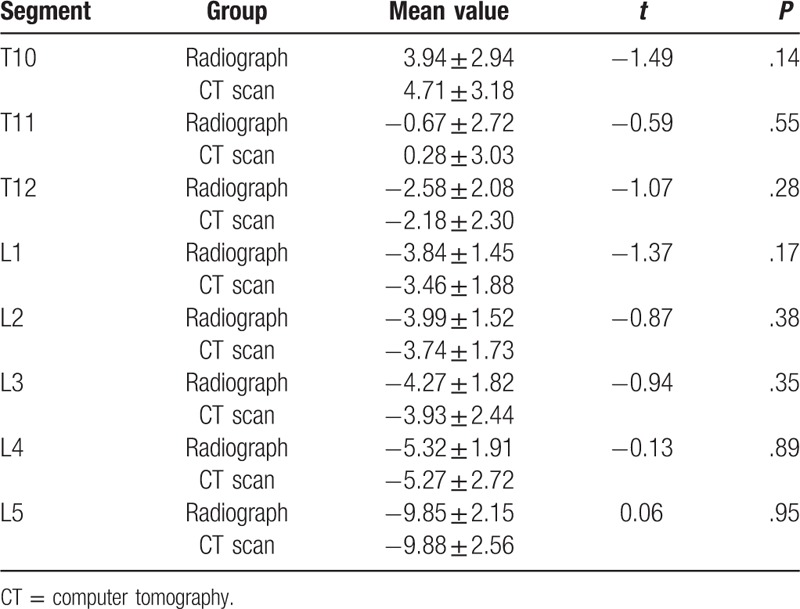
The distance between the superior margin of spinous process root and the lower edge of pedicle and comparison between radiographs and CT scans (mm *x* ± s).

For T10 to T12, the superior margin of SP root gradually approached the pedicle axis with a distance from 9.37 ± 2.01 mm to 4.09 ± 2.31 mm. For L1 to L4, the superior margin of SP root was almost at the same latitude of the pedicle axis, the distance ranged from 2.68 ± 2.15 mm to 0.21 ± 2.43 mm. For L5, the superior margin of SP root was above the pedicle axis, and the distance was 4.69 ± 2.02 mm. There were no statistical significance between CT scans and radiographs. (shown in Table 3)

**Table 3:**
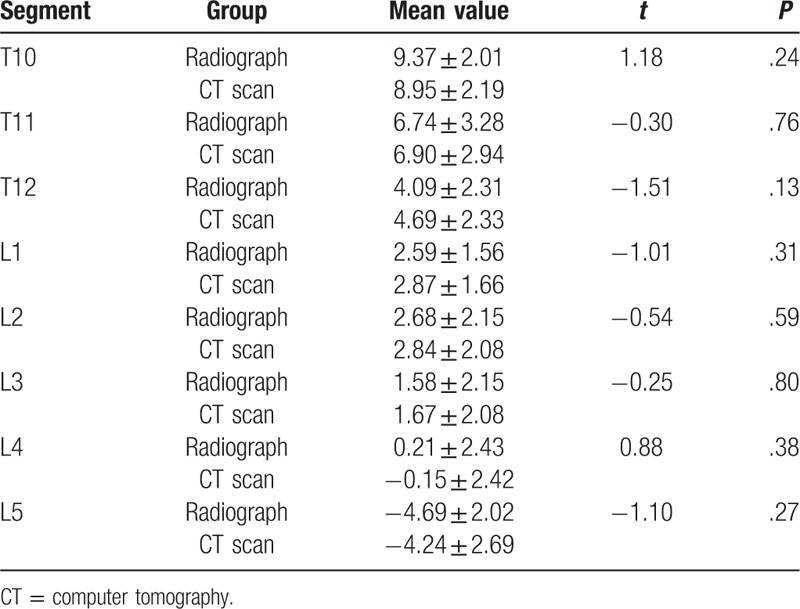
The distance between the superior margin of spinous process root and the pedicle axis and comparison between radiographs and CT scans (mm *x* ± s).

### 3.1 The pedicle screw placement simulating

**Figure 5:**
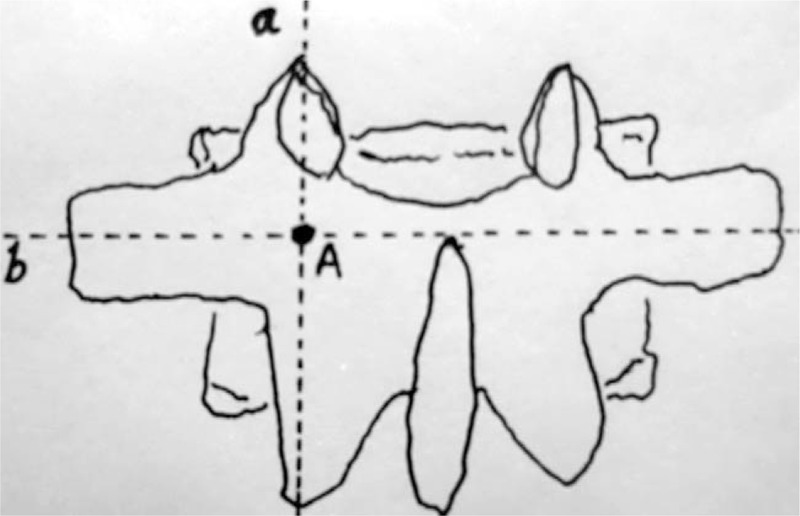
The diagram of the basic locating point of the spinous process locating method. (A) The central vertical line of zygapophysial joint. (B) The horizontal line of superior margin root of spinous process. Point A = the basic locating point.

According to the above data, the basic reference point was chosen at the intersection between the horizontal line of superior margin of SP root and the central vertical line of the facet joint (shown in Fig. 5). The screw entry points were above 5 mm of the basic locating point at T10 and T11. At T12, it was slightly above the basic reference point. Form L1 to L4, it was the basic reference point. At L5, it was 5 mm below reference the basic point. In total, 80 pedicle screws were placed in the pedicle at 5 specimens. There was no penetration of the pedicle cortex (shown in Figs. 3 and 4).

## 4 Discussion

The pedicle provides the strongest holding force to the attachment to the spine. Thus, compared with hooks or others kinds of instrumentation, a pedicle screw provides the best internal stability. Many instrumentation systems use a screw which enters pedicle and vertebral body for fixation. However, the pedicle screw maintains technically demanding due to the inherent anatomy. It might cause a number of complications. Misplacement is the commonest complication. Nerve root injury due to the misplaced pedicle screw is the devastating one.

To prevent such a complication, there are several methods proposed to accurately place the pedicle screw in a previous study. They use bony marks of vertebral laminar to locate the entrance point and then choose the direction and the depth. Roy-Cammille and Margel choose the transverse process and the facet joint as the bony marks. Du chooses the isthmus crest and the accessory process crest.

Roy-Cammille et al^[2,4]^ and Magerl^[5]^ located the entrance point at the intersection point between the lateral border of superior facet joint and the midline of transverse process. During the procedure of pedicle screw insertion, it requires excessive exposure of the transverse process. It causes major trauma, probably bleeding and potential neurological complications. It also requires long operation time. When transverse process fracture occurred, it would have made it difficult to locate the entrance point and then cause screw misplacement.

For the thoracic spine, some previous studies reported that the midline of transverse process is only close to the pedicle axis at T6 and T7. For the other thoracic segments, it is far from the pedicle axis.^[9]^ Therefore, the Magerl method is not applicable to the thoracic spine.^[5]^ Moreover, except at L4, the axis of lumbar pedicle is not at the midline of the transverse process.^[10]^

Du et al^[6]^ located the entrance point at the top joint where the isthmus crest conversed the accessory process crest. This method can only applicate in the lumbar spine. For the thoracic spine and sacrum, the accessory process does not exist. Thus, another locating method needs to be investigated in order to supplement the transverse process and the accessory process method.

The spinous process is a constant structure of spinal laminar. It is investigated as the locating mark of the pedicle screw entrance point in the study.

The superior margin of spinous process root at T12 to L5 is located within the range of the pedicle axis. It provides the prerequisite for the spinous process locating method. For L1 to L4, the superior margin of spinous process root is very close to the pedicle axis, mostly within 2 mm around the pedicle axis. For L5, it is located 5 mm beyond the axis.

The spinous process locating method does not need to expose the transverse process. The entrance point could be defined just with the spinous process and the facet joint exposed. It reduces the iatrogenic trauma, bleeding, and neurological complication. Meanwhile, the spinous process is relatively constant. For the patients with transverse process fracture, the spinous process locating method can still be applied.

The radiological method is often applied in the anatomical study, as well as the pedicle screw insertion navigating.^[11–13]^ It is easy to get the radiographs and CT scans before the operation. By them, we can measure the distance between the superior margin of spinous process root and the pedicle axis, as well as the diameter of the pedicle. According to the study, there was no significant difference between the radiographs and CT scans. It is much easier to obtain radiographs and fluoroscopy than the CT scans during the operation. The fluoroscopy is reliable during pedicle screw placement as well as needle insertion in percutaneous kyphoplasty (PKP). There are 2 key points to insert the pedicle screw under fluoroscopy.

The first one is to pay attention to the angle of screw insertion. The inward tilt angle is similar between the spinous process locating method and the transverse process locating method. It is needed to inward the screw end to the head in 5° to 10° when inserting, especially at the thoracic spine.

The second issue is the real anteroposterior fluoroscopy position of the vertebrae. On the fluoroscopy, the end plate of the vertebrae should coincide to 1 line. Due to the thoracic kyphosis and the lumbar lordosis, the C-arm machine position alters for every single vertebrae. If the bilateral sign was observed on the fluoroscopy, the C-arm machine should be rotated caudally or cranially. However, it is difficult to get the position when the vertebrae is fractured, especially during the percutaneous kyphoplasty. Under that condition, the vertebral body is compressed; thus, the endplate could not be coincided. It cannot be used to reveal the position of vertebrae.

The combination of the spinous process and transverse process locating method can be used to do so under the condition of vertebrae body fracture. The relationship of pedicle entrance and transverse process on the fluoroscopy is almost constant, because they are at the same plane. But the spinous process root is at the rear plane. On the real anteroposterior fluoroscopy of the vertebra, the midpoint of the transverse process root, the pedicle axis, and the superior margin of spinous process root will coincide at the same line. Otherwise, it is not the real anteroposterior fluoroscopy.

There are several pitfalls of the study. The first one is the limited case series. More cases will be more promoting significance. The second one is that the degenerative changes would also decrease the accuracy of this new method. The last one is that it cannot be used in several approaches, such as the Wiltse paraspinal approach. After all, it is a very helpful supplement to the existing methods to locate the pedicle screw entrance point.
